# Influence of yellow gypsum on nutrient uptake and yield of groundnut in different acid soils of Southern India

**DOI:** 10.1038/s41598-022-09591-1

**Published:** 2022-04-04

**Authors:** Laxmanarayanan M, Prabhudev Dhumgond, Shruthi, Jahir Basha C R, Supriya Sarkar, Prakash Nagabovanalli B

**Affiliations:** 1 Plant Nutrition Laboratory, Department of Soil Science and Agriculture Chemistry, University of Agricultural Sciences, GKVK, Bengaluru, Karnataka 560065 India; 2 Agricultural Research Station, Pavagada, Tumkur, Karnataka 561202 India; 3 Environmental Research Group, Tata Steel Limited, Jamshedpur, Jharkhand 831007 India

**Keywords:** Agroecology, Plant ecology

## Abstract

Yellow gypsum (YG), a synthetic product from Linz-Donawitz slag containing high iron (Fe) (5.41%), zinc (Zn) (0.37%) and silicon (Si) (3.41%) can be used as a source of these nutrients along with calcium (Ca) and sulphur (S) for groundnut production. Three field experiments were conducted to know the effect of different rates (500 and 625 kg YG ha^−1^) and time of application (basal alone and basal + split) of YG on growth, yield and economic returns of groundnut, and micronutrient and Si availability and their uptake in comparison with basal application of 500 kg natural gypsum (NG) ha^−1^. Basal alone and basal + split application of YG significantly increased the growth, yield and economic returns of groundnut. Further, it increased the soil pH, availability of micronutrients, Si and their uptake by haulm and kernel of groundnut over NG. Irrespective of the location, YG application recorded higher plant available nutrient (PAN) coefficient of micronutrients, while NG application recorded higher PAN recovery coefficient of Si. Basal + split application of YG resulted in better growth and yield of groundnut than basal application of YG. In conclusion, YG can be a potential alternative for NG as a source of Fe, Zn and Si along with Ca and S for groundnut production.

## Introduction

In India, intensive agriculture with high yielding and fertilizer responsive wheat and rice varieties to increase the food grain production led to deplete the soil nutrient resources to an extent which could not sustain the productivity. Subsequently, agricultural soils of India have a general calculated annual nutrient (N + P_2_O_5_ + K_2_O) deficit of about 10 million tonnes (Mt)^1^. It was also projected that this nutrient gap may widen to 22 Mt in 2025 at an overall nutrient consumption of 350 Mt^2^. Besides macronutrients, deficiency of micronutrients is widespread in Indian soils, and it is a major constraint in achieving higher crop production^3^. A recent analysis of soil samples across the country showed that 43.40%, 14.40%, 6.10% and 7.90% are deficient in zinc (Zn), iron (Fe), copper (Cu) and manganese (Mn), respectively^3,4^. This was mainly attributed to cultivation of high productive crops with continuous exportation of micronutrients without proper replenishment, imbalanced application of fertilizer nutrients^5^, considerable decrease in recycling of crop residues and inadequate application of bulk manures^6^. Further, lower application of micronutrient fertilizers due to high-cost per unit nutrient application and non-existence of fertilizer sources which supplies micronutrients along with primary and secondary nutrients has also resulted in micronutrient deficiencies in soil^7^. Widespread micronutrient deficiency and higher cost of micronutrient fertilizer emphasizes the need for more research to find fertilizers which can be cost effective and supply micronutrients along with major and secondary nutrients.

Next to soybean, groundnut shares 27% and 25% of total oil seed and vegetable oil production in India, respectively^8^. It is generally grown in well drained coarse textured soil with high fertility and 6.0–7.5 pH. Groundnut being an oil seed and legume crop, its calcium (Ca) and sulphur (S) requirement is high^9^. Calcium and sulphur plays several crucial roles in growth and development of groundnut such as increasing seed oil and protein content, pod and haulm yield, formation of sulphur containing amino acids and synthesis of chlorophyll and vitamins^10^. However, micronutrient cations being a metal ions act as metal component and regulatory co-factor of a large number of enzymes^11^. Iron functions in plants are enzyme activation, chlorophyll formation and nitrogen fixation^12^. Zinc involves in many crucial plant functions such as activation of enzymes and biosynthesis of growth substances such as auxin^13^. Silicon (Si) is a second most abundant element in the earth crust and it is recognized as beneficial element for plant growth^14^. Effect of Si on alleviation of biotic and abiotic stress such as disease and pest, drought, salinity and metal toxicity in various Si accumulating and non-accumulating species has been widely reported^15^. Though Si content is low in legumes, recent studies have showed positive effect on growth and yield of soybean^16^, alfalfa^17^ and cowpea^18^. Application of gypsum as source of Ca and S for groundnut cultivation is common practice^10^. However, continuous dependence on natural gypsum increase the stress on natural gypsum deposits which are major raw material for commercial gypsum production. Therefore, gypsum produced from industrial waste can be useful for effective conservation of natural gypsum deposits.

Yellow gypsum (YG) is a synthetic gypsum produced from Linz-Donawitz (LD) slag by treating with concentrated sulphuric acid and neutralized with lime. Besides Ca (23.03%) and S (17.48%), YG contains ample amount of Fe (5.41%), Zn (0.37%) and Si (3.41%) and, meager amount of Mn (0.09%) and Cu (0.0004%)^19^. YG is an industrial waste-based value-added nutrient supplement and could be a promising and cost-effective source of Fe, Zn and Si along with Ca and S. In general, gypsum solubility and availability of Ca and S is controlled by its particle size and soil moisture condition. Hence, application of 50% of gypsum at planting and another 50% at peg initiation stage could be the best alternative method of application for groundnut production^5^. Further, application of gypsum as basal and split enables plants to take up applied nutrients more efficiently^20^. By keeping that into consideration, in the present study YG was applied at both planting and peg initiation stage of groundnut.

The positive effect of natural gypsum on groundnut productivity and availability of Ca and S in soils is well studied, but, utilization of yellow gypsum made from LD-slag as source of Fe, Zn and Si along with Ca and S is not studied. Further, its effect on productivity of groundnut and availability of micronutrients and Si in different acid soils is least known. Therefore, an attempt has been made to study the influence of yellow gypsum on soil micronutrients and silicon and their uptake and productivity of groundnut in different acid soils of Southern India. Further, this study also aims to evaluate the comparative effect of various rates and time of application of YG against basal application of NG.

## Materials and methods

### Description of the experimental sites

Three field experiments were established at two major groundnut growing areas of Karnataka in Baljigapade, Chikkaballapur district (N 13° 26′ 73.5″, E 77° 46′ 59.2″ with an elevation of 915 m above mean sea level) and Pavagada, Tumkur district (N 14° 07′ 59.7″, E 77° 16′ 34.6″ with an elevation of 768 m above mean sea level). In Baljigapade, two field experiments were conducted during 2018 and 2019 at Agricultural Research Station (ARS), where in Pavagada, a field experiment was conducted during 2018 at farmer’s field. All the experiments were performed in accordance with relevant guidelines and regulations.

#### Soil characteristics and climatic conditions

**Figure 1:**
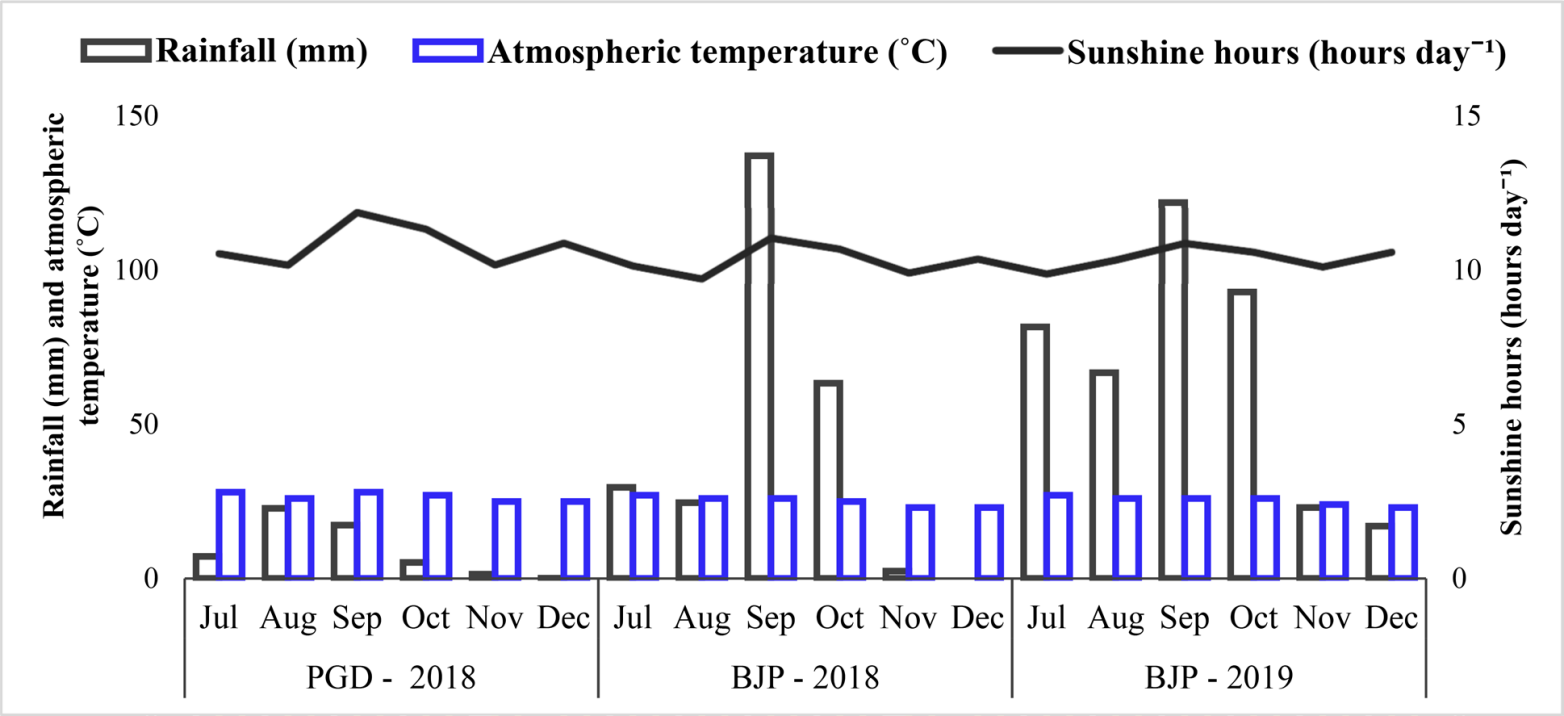
Mean monthly rainfall (mm), atmospheric temperature (°C) and sunshine hours (hours day^−1^) at Pavagada (PGD) during 2018 and Baljigapade (BJP) during 2018 and 2019. The groundnut growing period was early July to early December at all the locations.

Soil Texture of both the experimental plots in Baljigapade was sandy clay loam in nature and belongs to the taxonomical suborder *kandic paleustalfs*, and falls under Eastern Dry Agro-Climatic Zone. The soil of the Pavagada experimental site is a sandy loam of the taxonomical suborder *chromic haplusterts*, and belongs to the Central Dry Agro-Climatic Zone. Monthly mean rainfall (mm), temperature (°C) and sunshine hours (hours day^−1^) of all three locations in 2018 and 2019 were collected from local weather stations located near experimental sites at Chikkaballapur and Pavagada (Fig. 1). Prior to experiment, surface soil samples (0–15 cm) of all the locations were collected and physicochemical properties were determined by following standard analytical methods (Table 1). pH of Pavagada soil was neutral, whereas Baljigapade soils in 2018 and 2019 were moderately and very strongly acidic, respectively.

**Table 1:** Initial soil characteristics of experimental sites.

Soil parameters	Pavagada (2018)	Baljigapade (2018)	Baljigapade (2019)
Soil reaction (1:2.5 soil: water ratio)	6.93	5.77	4.80
EC (m Sm^−1^)	1.40	5.90	8.94
Clay (%)	15.90	28.71	27.40
Silt (%)	7.60	9.27	15.30
Sand (%)	76.50	62.02	56.20
Soil texture	Sandy loam	Sandy clay loam	Sandy clay loam
DTPA-extractable Fe (mg kg^−1^)	5.03	16.35	16.18
DTPA-extractable Mn (mg kg^−1^)	0.25	1.57	2.25
DTPA-extractable Zn (mg kg^−1^)	1.28	1.42	0.72
DTPA-extractable Cu (mg kg^−1^)	0.43	1.36	1.42
0.5 M acetic acid Si (mg kg^−1^) (AA-Si)	51.75	41.50	15.96
0.01 M CaCl_2_ Si (mg kg^−1^) (CC-Si)	45.06	43.16	21.61

Each value represents the mean value of three replications.

### Crop establishment and management

Prior to the establishment of the experiments, land was prepared by ploughing and levelling. After levelling, 5 × 4 m size plots were formed manually and the recommended dose of fertilizer was given at the rate of 25 kg N ha^−1^, 75 kg P_2_O_5_ ha^−1^ and 37.5 kg K_2_O ha^−1^ as urea, diammonium phosphate and muriate potash, respectively. Applied dose of fertilizer in the present study has been adopted from the package of practice (POP) for groundnut cultivation in Karnataka state as recommended by University of Agricultural Sciences, Bangalore, India. The entire dose of nitrogen, phosphorous and potassium were applied as basal during sowing of the groundnut. In Baljigapade experimental plots, dibbling was done on 9 August 2018 and 7 August 2019 where in Pavagada, it was on 20 July 2018. Spanish bunch type variety Kadiri—6 (selected from the local cultivars and complying with relevant institutional, national, and international guidelines and legislation)^21^ was used as test crop. Seeds were sown by adopting 30 × 10 cm spacing and field was irrigated immediately after sowing. The crop was harvested on 2 December 2018 and 2 December 2019 at Baljigapade, on 10 November 2018 at Pavagada.

### Experimental design and treatments

The field experiments were laid out in a randomized block design with three replications. Two gypsum sources namely YG and NG were used. The YG was provided by Tata Steel Ltd., Jamshedpur, Jharkhand, India. The NG is locally available mined gypsum and procured from Argyan Bio-tech, Tumkuru at Pavagada. Finely grounded 0.1 g gypsum materials were predigested with 7:2:1 ratio of nitric acid, hydrogen peroxide and hydrogen fluoride. Later, predigested samples were digested using microwave digester (Milestone- START D). The digested samples were analyzed by using ICP–OES (Thermofisher) and the chemical composition of YG and NG is presented in Table 2.

**Table 2:** Chemical composition of yellow gypsum and natural gypsum.

Parameters	pH (1:100 gypsum: water ratio)	Ca	SO_4_^2−^	P_2_O_5_ (%)	Fe (%)	Mn (%)	Zn (%)	Cu (%)	SiO_2_ (%)
		(in % by mass)							
Yellow gypsum	8.14	23.03	17.48	0.32	5.41	0.09	0.37	0.0004	3.41
Natural gypsum	4.92	23.13	17.95	NA	0.03	0.002	0.004	0.0015	1.37

NA—Not available. Each value represents the mean value of three replications.

Gypsum sources were powdered and passed through 2 mm sieve and used in the study. YG was applied at different time as basal and basal + split, whereas NG was applied as basal alone. Using two gypsum sources, totally five treatments were formulated: two rates of YG (500 and 625 kg ha^−1^) applied at different time (Basal alone and basal + split) while 500 kg NG ha^−1^ was used as control which has been adopted in the package of practice for groundnut recommended by University of Agriculture Sciences, Bangalore, India. Basal + split application treatments received 50% of YG at sowing and remaining 50% at peg initiation stage while basal application of YG and NG treatments received entire dose at sowing. For basal application, gypsum sources were manually broadcasted before sowing, while for basal + split application, YG was band placed near the established plants.

### Experimental measures

#### Growth components of groundnut

Plant height and number of branches were recorded on five tagged plants in the middle rows at harvest. The values of plant height and number of branches are given as mean of five plants from each plot.

#### Yield and yield components of groundnut

To measure pod and haulm yield in each plot, plants from a four center rows were harvested manually and dried in an oven at 65–70 °C for 3–5 days until the weight was constant. The collection of groundnut plants complied with guidelines in Karnataka state and regulations in India. Harvest index was calculated by taking the ratio of dry pod yield and total above ground biomass yield. The number of pods plant^−1^ were determined by counting the pods in tagged plants in the middle rows. To determine the 100 kernels weight, pods were removed from randomly selected plants and shelled manually and it was determined on one hundred kernels randomly sampled from shelled kernels. Shelling percentage was calculated as the ratio of kernel weight and pod weight and expressed as percentage.

#### Soil sampling and chemical analysis

A representative post-harvest composite samples (0–15 cm) were collected from each treatment plot at harvest and then samples were air dried, powdered and passed through 2 mm sieve for analysis of soil chemical properties. Soil texture was measured by International pipette method^22^. Soil pH and electrical conductivity (EC) of the samples were determined through glass electrode pH meter and a conductivity meter, respectively by taking 1:2.5 soil water suspensions^23^. To determine Diethylene triamine penta acetic acid (DTPA) extractable Fe, Mn, Zn and Cu, soil was extracted with 0.005 M DTPA + 0.1 M TEA + 0.01 M CaCl_2_ (pH 7.3) and their contents were determined using atomic absorption spectrophotometer^24^ (PerkinElmer PinAAcle™ 900F, USA). The plant-available Si was extracted by using 0.01 M CaCl_2_^25^ and 0.5 M acetic acid^26^ and determined by molybdenum blue colorimetric method.

#### Calculation of plant available nutrient (PAN) recovery coefficient and nutrient uptake

The relationship between micronutrients and silicon quantity in the soil and its uptake by groundnut were determined. The plant available nutrient recovery coefficient was calculated using formula,

$${\text{PAN recovery coefficient }} = \frac{{{\text{Nutrient uptake with plant yield }}\left( {{\text{g ha}}^{{ - {1}}} } \right)}}{{{\text{Nutrient quantity in soil }}\left( {{\text{g ha}}^{{ - {1}}} } \right)}}$$

The PAN recovery coefficient expresses the relation between plant nutrient uptake and quantity of nutrient available in the soil (0–15 cm layer)^27,28^. Numerical values of micronutrients and silicon provides information on how many times micronutrients and silicon were smaller or greater than their quantity in soil. At PAN recovery coefficient values < 1.0, micronutrient and silicon quantity in soil are sufficient for plant nutritional needs. When PAN recovery coefficient values > 1.0, the need of plants with regards to micronutrients and silicon exceeds their quantity in the soil.

Nutrient uptake by haulm and kernel of groundnut was calculated using formula,

$${\text{Nutrient uptake }}\left( {{\text{g ha}}^{{ - {1}}} } \right) = \frac{{{\text{Nutrient concentration }}\left( \% \right) \, \times {\text{ Biomass }}\left( {{\text{kg ha}}^{{ - {1}}} } \right)}}{100}$$

#### Plant sampling and analysis

For analysis, ten tagged plant samples were randomly selected from each plot at harvest stage and oven dried at 60 °C until attaining a constant weight. After drying, plant samples were powdered and used for analysis. Powdered plant sample (0.1 g) was predigested with 7 ml HNO_3_ and 3 ml H_2_O_2_ in PTFE (Poly Tetra Fluoro Ethylene) vessels and later digested using a microwave digester (Milestone—START D). The digested samples were made volume up to 50 ml using deionized water. Content of micronutrients in digested samples of haulm and kernel were determined using atomic absorption spectrophotometer (PerkinElmer PinAAcle™ 900F, USA). For silicon, plant samples were pre- digested with 7 ml HNO3 (70%), 2 ml H2O2 (30%) and 1 ml HF (40%) and later digested using a microwave digester and digested sample made volume up to 50 ml using 4% boric acid. Content of silicon in digested samples of haulm and kernel was determined by the colorimetric molybdenum blue method at 600 nm^29^ using UV–Visible spectrophotometer (SHIMADZU Pharma spec, UV-1700 series) with auto sample changer (ASC-5).

#### Economic analysis

Economic analysis was performed to assess the economic feasibility of different sources, rates and times of YG and NG application. Land rent, seedbed preparation, seeds, fertilizers, sowing, crop protection measures and harvesting were considered as fixed costs. Further, price of gypsum materials and labor charges incurred on different application times are included in variable cost. Total cost was calculated based on existing prices of groundnut pod and haulm. Net income (US$) was computed by subtracting the total expenditure from gross income. The benefit cost ratio was computed by dividing net income with total expenditure.

### Statistical analysis

A two-way analysis of variance (ANOVA) was performed to analyze the effect of different treatments on the soil physiochemical properties, available nutrients at post-harvest soils, yield and growth parameters of groundnut and nutritional status (uptake of nutrients by haulm and kernel of groundnut, and PAN recovery coefficient). Least significant difference (LSD) test was used to evaluate the significant differences between the treatments. The small letters in tables and figures indicated statistically significant differences at *P* < 0.05. All the figures were plotted by R programme^30^.

### Statement in the collection of plant material

The collection of Groundnut is in compliance with guidelines in Karnataka state and regulations in India. All collection was done with the permission of the relevant regulatory governing bodies and with reference to the relevant legislation.

## Results

### Seasonal conditions during crop growth

The groundnut crop produces optimum yield in the regions receiving rainfall between 200 to 1000 mm^8^. The total rainfall during groundnut growing season was 257.10 mm and 403.10 mm at Baljigapade in 2018 and 2019, respectively, wherein Pavagada (2018) total rainfall was 53.90 mm (Fig. 1). In 2018, both Pavagada and Baljigapade received very low and negligible rainfall during the reproduction and harvest stage of groundnut. Optimum temperature for groundnut production ranges between 20 to 30 °C and growth and pod formation limited below 16 °C and above 32 °C^9^. The monthly mean atmospheric temperature was ranged from 23 to 27 °C and 21 to 26 °C at Baljigapade in 2018 and 2019, respectively, wherein Pavagada ranged from 25 to 28 °C. At all three locations, the monthly mean atmospheric temperature was slightly high during the early vegetative growth of groundnut and it was progressively decreased as the crop reaches its maturity stage (Fig. 1). All three locations recorded higher and lower mean monthly sunshine hours (hours day^−1^) during peg initiation to pod filling stage (September and October) and early vegetative growth of groundnut (July and August), respectively.

### Growth parameters of groundnut

**Figure 2:**
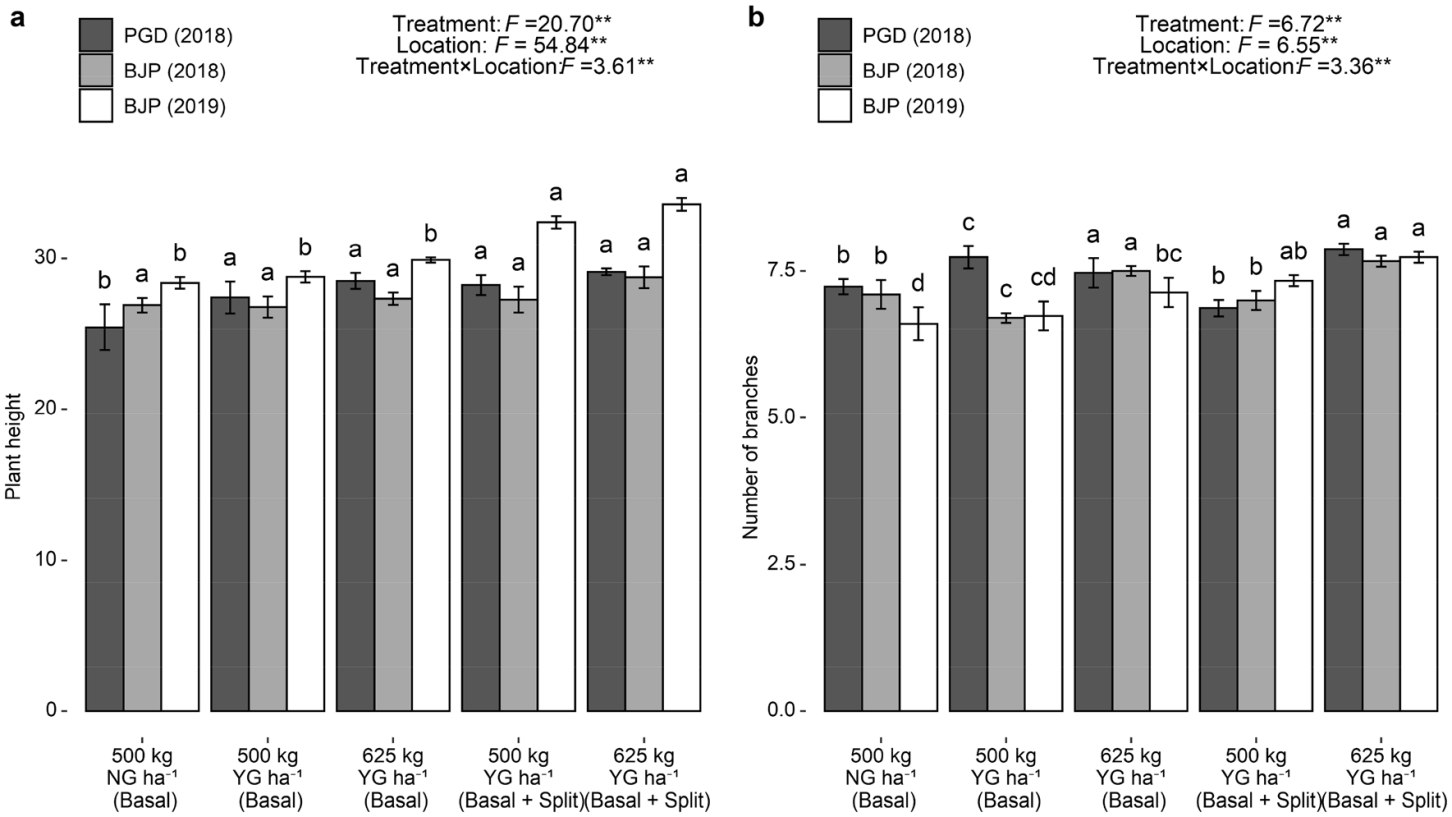
Effect of YG and NG on plant height (**a**) and number of branches (**b**) of groundnut. F value and significance level (**P < 0.01, *P < 0.05 and ^ns^*P* ≥ 0.05). Values followed by a different letter within the same location are significantly different at *P* < 0.05 probability level. Capped bars at the surface of vertical bars represent the standard deviation, *n* = 3. *YG* yellow gypsum, *NG* natural gypsum, *PGD* Pavagada, *BJP* Baljigapade.

Analysis of variance revealed that treatment, location, and their interaction had a significant effect on plant height and number of branches at harvest (P < 0.01) (Fig. 2a,b). Application of gypsum at different rates resulted in a significant difference in the plant height and number of branches at all three locations except on plant height at Baljigapade during 2018. Irrespective of the locations, higher and lower plant height and number of branches was observed with treatment receiving 625 kg YG ha^−1^ as basal + split and 500 kg NG ha^−1^ as basal, respectively. With respect to different times of YG application, plant height was not significantly influenced, except at Baljigapade in 2019. However, basal + split application treatments recorded a significantly higher number of branches over basal application treatments.

### Yield and yield components of groundnut

Yield components of groundnut were significantly affected by different treatments and locations. Further, there was no significant difference observed due to its interaction (Table 3). Application of YG at different rates significantly affected the yield components of groundnut at all three locations, except 100 kernel weight and shelling percentage at Pavagada and Baljigapade in 2018, respectively. Significantly higher yield components were recorded with basal and basal + split application of 625 kg YG ha^−1^ and lower yield components were recorded with control. Regardless of the locations, application of YG at different times had no significant effect on the number of pods plant^−1^. However, significant differences in 100-kernel weight and shelling percentage were observed at Baljigapade in 2019.

**Table 3:** Effect of different sources, time and rate of application of gypsum application on yield and yield components of groundnut.

Locations and year of experiment	Gypsum sources	Time and rate of application (kg ha^−1^)		Yield components			Yield (kg ha^−1^)		
		Basal	Split	Number of pods plant^−1^	100 kernel weight (g)	Shelling percentage (%)	Pod yield	Haulm yield	Harvest Index (HI)
Pavagada 2018	NG	500	–	25.73 c	29.00 a	68.61 c	2487.40 d	5052.53 b	0.49 a
	YG	500	–	27.37 bc	29.33 a	70.37 bc	2598.00 bc	5480.05 ab	0.48 a
		625	–	28.27 ab	30.33 a	71.51 ab	2653.97 b	5824.11 ab	0.46 a
		250	250	28.00 b	28.33 a	71.65 ab	2542.92 cd	5913.13 a	0.43 a
		312.5	312.5	30.07 a	31.67 a	73.62 a	2753.91 a	5985.36 a	0.46 a
Baljigapade 2018	NG	500	–	25.80 c	27.66 c	70.42 a	2526.27 c	4935.94 b	0.51 a
	YG	500	–	27.07 bc	29.00 bc	72.54 a	2571.79 c	4613.91 c	0.56 a
		625	–	28.27 ab	29.66 ab	73.42 a	2742.80 ab	5158.02 ab	0.53 a
		250	250	27.93 ab	28.33 bc	72.22 a	2604.00 bc	5135.82 ab	0.51 a
		312.5	312.5	29.17 a	31.35 a	74.26 a	2798.33 a	5280.01 a	0.53 a
Baljigapade 2019	NG	500	–	24.63 c	28.38 b	65.43 c	2491.95 c	4616.49 b	0.54 a
	YG	500	–	25.27 c	28.60 b	66.00 bc	2503.05 bc	4667.55 b	0.54 a
		625	–	28.43 a	31.14 a	68.44 bc	2675.10 ab	4876.23 ab	0.55 a
		250	250	26.30 bc	30.95 a	69.13 b	2608.75 bc	4963.92 ab	0.53 a
		312.5	312.5	27.77 ab	31.30 a	72.41 a	2808.75 a	5345.76 a	0.53 a
*F* value	Treatment			11.20**	5.89**	9.13**	16.41**	10.62**	1.68^ns^
	Location			5.20*	1.24^ns^	19.03**	1.01^ns^	39.38**	25.68**
	Treatment × location			0.53*	0.80*	0.69*	0.50*	1.21*	0.74*

F value and significance level (**P < 0.01, *P < 0.05 and ^ns^P ≥ 0.05). Values followed by a different letter within the same column and the same year are significantly different at P < 0.05 probability level. *YG* yellow gypsum, *NG* natural gypsum.

Analysis of variance showed that different treatments significantly affected the pod and haulm yield, while different locations significantly affected the haulm yield and harvest index (Table 3). The treatment and location interaction had no significant effect on pod and haulm yield and harvest index of groundnut. YG receiving treatments recorded increase in pod yield over control, which ranged 1.08–10.76% at Pavagada (2018), wherein Baljigapade it ranged 10.76–1.80% and 12.71–0.44% in 2018 and 2019, respectively. Application of YG at different time had no significant effect on pod yield at Baljigapade, however, at Pavagada basal + split application of 625 kg YG ha^−1^ (2753.91 kg ha^−1^) recorded significantly higher pod yield than the basal application of 625 kg YG ha^−1^ (2653.97 kg ha^−1^).

The pod yield of groundnut was significantly and positively correlated with total uptake of Fe (*r* = 0.95*), Mn (*r* = 0.99**), Zn (*r* = 0.96*), Cu (*r* = 0.93*), and Si (*r* = 0.96*) by crop (see Supplementary Fig. S1 online). The haulm yield was significantly different among treatments at all three locations and recorded higher with YG receiving treatments than in NG receiving treatments. Haulm yield at Pavagada (2018) and Baljigapade (2018 and 2019) was increased by 11.84%, 6.97%, and 15.71%, respectively, in the basal + split application of 625 kg YG ha^−1^ compared with NG. Haulm yield of groundnut was significantly and positively correlated with total uptake of Fe (*r* = 0.92*), Mn (*r* = 0.88*), Cu (*r* = 0.97**) and Si (*r* = 0.96**) (see Supplementary Fig. S1 online). Irrespective of the locations, the harvest index of groundnut was not significantly affected by application of YG at different rates and time.

### Changes in physiochemical properties of post-harvest soil

**Figure 3:**
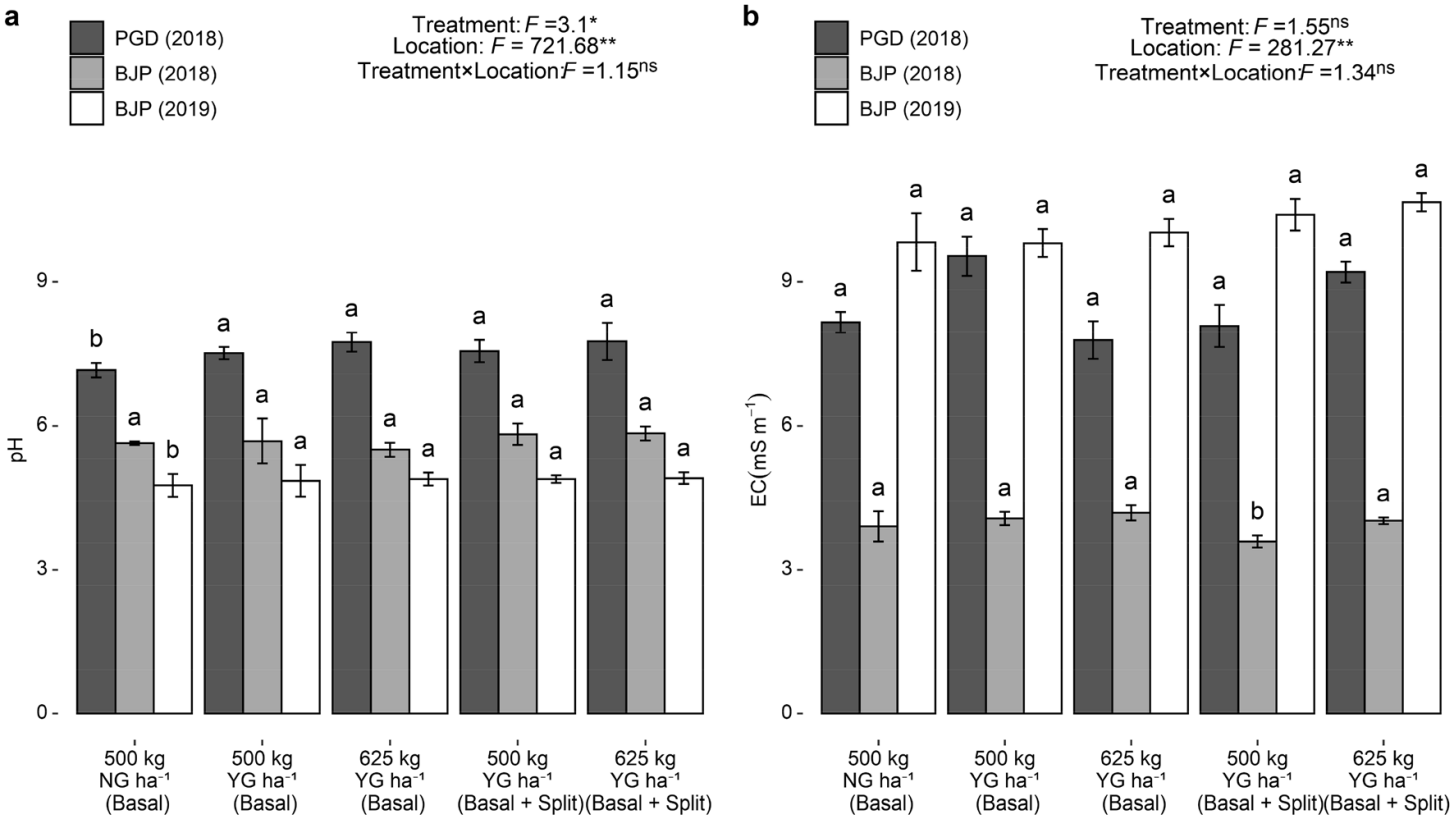
Effect of YG and NG on pH (**a**) and electrical conductivity (EC) (**b**) of post-harvest soils of PGD (2018) and BJP (2018 and 2019). F value and significance level (**P < 0.01, *P < 0.05 and ^ns^*P* ≥ 0.05). Values followed by a different letter within the same location are significantly different at *P* < 0.05 probability level. Capped bars at the surface of vertical bars represent the standard deviation, *n* = 3. *YG* yellow gypsum, *NG* natural gypsum, *PGD* Pavagada, *BJP* Baljigapade.

Post-harvest soil pH differed significantly among different treatments and locations, while post-harvest soil electrical conductivity (EC) varied significantly among different locations (Fig. 3a,b). Application of various levels of YG at different times over control had no significant effect on post-harvest soil pH at Baljigapade in 2018. Nevertheless, there was a significant increase in the other two locations. In both Pavagada (2018) and Baljigapade (2019), all the YG receiving treatments observed higher post-harvest soil pH than NG receiving treatment. The application of 625 kg YG ha^−1^ as basal + split increased the pH of Pavagada soil from 6.93 to 7.76 and from 5.77 to 5.84 and 4.76 to 4.92 in Baljigapade soils in 2018 and 2019, respectively. There was no significant difference among treatments with different time of YG application. Among the locations, Pavagada showed a remarkable change in pH at harvest while the other two locations showed a meager level of change in pH with YG application. Significant changes in EC with YG application were observed at Baljigapade during 2018, but it was not observed at other locations. EC of Pavagada soil ranged from 8.15 to 9.20 mS m^−1^ and that of Baljigapade soils ranged from 3.91 to 4.02 mS m^−1^ and 9.83 to 10.66 mS m^−1^ in 2018 and 2019, respectively. Further, there was no significant difference in EC among different times of YG application and different sources of gypsum.

### Effect of YG application on soil micronutrient and silicon status

Treatments, locations, and their interaction had significant effects on the DTPA extractable Fe, Mn, Zn, and Cu contents in post-harvest soils of all the locations (P < 0.01) (Table 4). The DTPA extractable Fe, Mn, Zn, and Cu contents in post-harvest soils were significantly varied with different rates of YG application, except DTPA extractable Cu at Pavagada in 2018. DTPA extractable Fe content in soils varied from 5.34 to 24.63 mg kg^−1^, with the highest in basal + split application of 625 kg YG ha^−1^ and lowest in control. Overall, DTPA extractable Fe content in soils increased with increasing YG rates, and all the YG receiving treatments recorded significantly higher DTPA extractable Fe content than NG treatment. DTPA extractable Mn content was higher with basal alone and basal + split application of 625 kg YG ha^−1^ than in the other treatments. Significantly higher DTPA extractable Zn was noted in the basal + split application of 625 kg YG ha^−1^ and all the YG receiving treatments recorded significantly higher DTPA extractable Zn than control, expect at Baljigapade in 2018. Significant increase in DTPA extractable Cu was observed with increasing rate of YG, with the higher content in 625 kg YG ha^−1^ as basal and basal + split application. Basal + split application of YG recorded higher DTPA extractable Fe, Mn, Zn and Cu contents than basal application of YG, but the difference was not significant, except for DTPA extractable Fe and Zn at Baljigapade and Pavagada in 2018, respectively. Overall, higher DTPA extractable Fe (24.63 mg ha^−1^), Mn (2.74 mg ha^−1^) and Cu (1.61 mg ha^−1^) in post-harvest soil was recorded with basal + split application of 625 kg YG ha^−1^, while higher DTPA extractable Zn (1.98 mg ha^−1^) was recorded with basal application of 625 kg YG ha^−1^.

**Table 4:** Effect of different sources, time and rate of application of gypsum on DTPA—extractable micronutrients and silicon in post-harvest soils of Pavagada (2018) and Baljigapade (2018 and 2019).

Locations and year of experiment	Gypsum sources	Time and rate of application (kg ha^−1^)		Fe	Mn	Zn	Cu	Si (mg kg^−1^)	
		Basal	Split	(mg kg^−1^)				CC-Si	AA-Si
Pavagada 2018	NG	500	–	5.34 c	0.24 d	1.15 c	0.41 a	36.65 c	45.21 b
	YG	500	–	6.63 ab	0.41 bc	1.25 b	0.44 a	48.88 b	55.33 a
		625	–	6.79 a	0.47 a	1.30 b	0.44 a	51.27 a	57.83 a
		250	250	6.60 ab	0.37 c	1.41 a	0.47 a	51.91 a	55.33 a
		312.5	312.5	6.81 a	0.44 ab	1.38 a	0.45 a	52.39 a	56.58 a
Baljigapade 2018	NG	500	–	16.80 d	1.55 b	1.59 b	1.24 b	26.65 d	35.13 d
	YG	500	–	21.52 bc	1.55 b	1.65 b	1.31 b	35.05 c	36.25 d
		625	–	23.17 ab	1.90 a	1.98 a	1.52 a	38.91 b	40.50 c
		250	250	23.80 a	1.95 a	1.59 b	1.32 b	37.29 b	45.00 b
		312.5	312.5	24.63 a	1.83 a	1.71 b	1.48 a	41.21 a	48.38 a
Baljigapade2019	NG	500	–	20.21 b	2.32 c	0.78 c	1.58 ab	23.72 c	17.83 a
	YG	500	–	22.28 a	2.40 bc	0.94 b	1.49 c	25.23 b	19.04 a
		625	–	23.10 a	2.64 ab	1.05 ab	1.58 ab	25.43 b	19.92 a
		250	250	22.84 a	2.49 abc	0.99 ab	1.54 b	25.97 ab	19.29 a
		312.5	312.5	23.38 a	2.74 a	1.09 a	1.61 a	26.78 a	20.79 a
*F* value	Treatment			67.31**	3.14*	19.58**	8.26**	199.49**	40.92**
	Location			3377.66**	4109.31**	409.08**	1896.60**	2247.77**	1680.02**
	Treatment × location			14.13**	2.39*	5.76**	4.64**	31.92**	11.64**

F value and significance level (**P < 0.01, *P < 0.05 and ^ns^P ≥ 0.05). Values followed by a different letter within the same column and the same year are significantly different at P < 0.05 probability level.

*YG* yellow gypsum, *NG* natural gypsum, *CC-Si* CaCl_2_ extractable Si, *AA-Si* acetic acid extractable Si.

Different treatments, locations and their interaction had significant effect on the acetic acid (AA-Si) and calcium chloride (CC-Si) extractable Si content in post-harvest soil (P < 0.01) (Table 4). Both, AA-Si and CC-Si were found to be significantly increased with increasing rate of YG, however, AA-Si at Baljigapade in 2019 was found to be non-significant. Application of 625 kg YG ha^−1^ as basal + split recorded significantly higher CC-Si in all locations, where lowest CC-Si was recorded in control. In all the locations, basal + split applications of YG recorded significantly higher CC-Si than basal application of YG. At both the locations in 2018, significantly higher AA-Si was observed with basal + split application of 625 kg YG ha^−1^. Unlike CC-Si, AA-Si was found to be non-significant among basal and basal + split application of YG treatments. In different treatments, CC-Si ranged from 23.72 to 52.39 mg kg^−1^, while AA-Si ranged from 19.04 to 56.58 mg kg^−1^. The pH of post-harvest soil was significantly and positively correlated with availability of AA-Si (*r* = 0.98**) and CC-Si (*r* = 0.96**) (see Supplementary Fig. S1 online). In general, YG applied treatments recorded significantly higher AA-Si and CC-Si than NG and Pavagada recorded higher CC-Si and AA-Si than Baljigapade in 2018 and in 2019.

### Micronutrients and silicon uptake by groundnut in relation to their availability in soil

**Figure 4:**
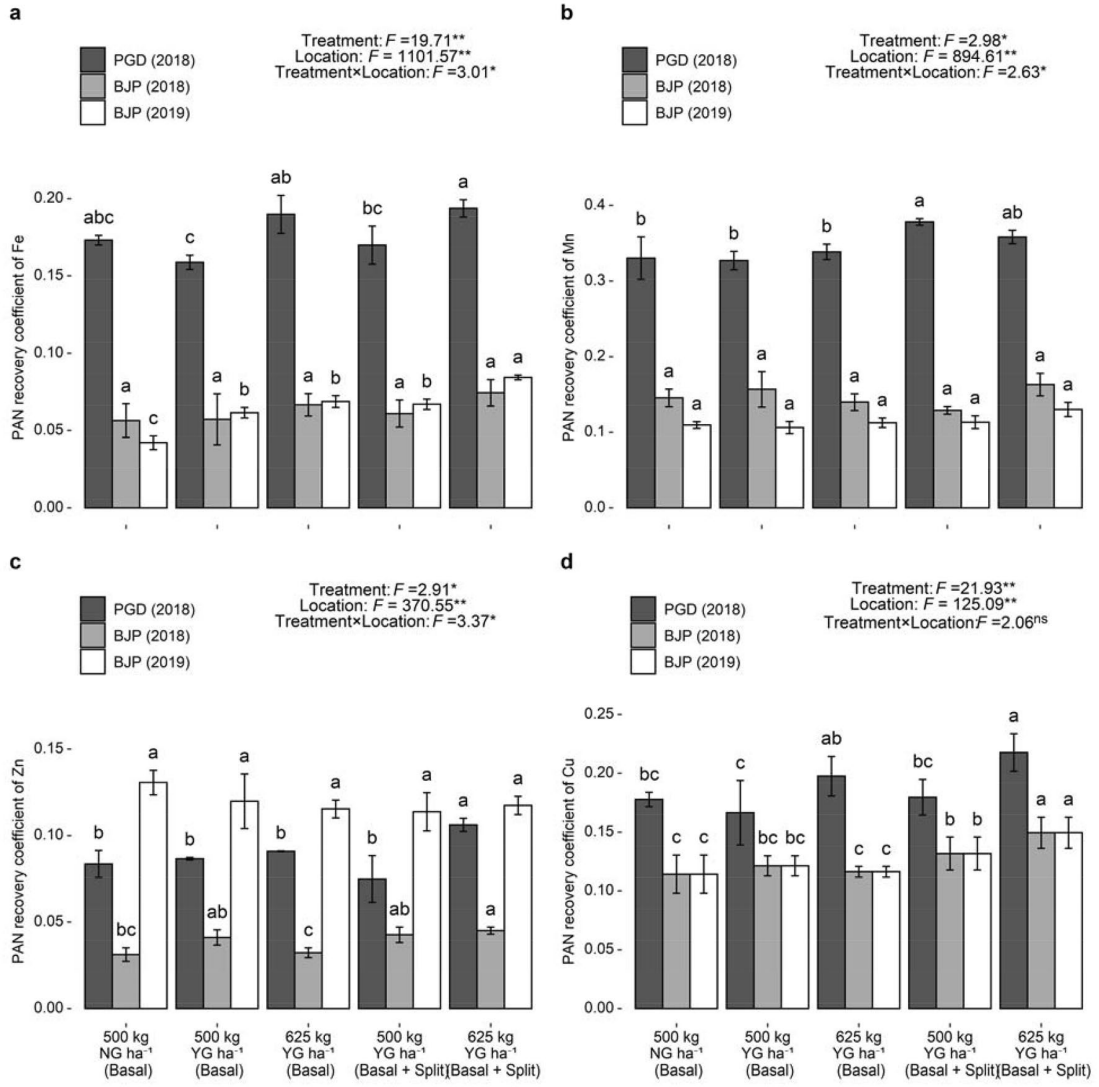
Plant available nutrient (PAN) recovery coefficient of Fe (**a**), Mn (**b**), Zn (**c**) and Cu (**d**) in groundnut under different YG treatments. F value and significance level (**P < 0.01, *P < 0.05 and ^ns^*P* ≥ 0.05). Values followed by a different letter within the same location are significantly different at *P* < 0.05 probability level. Capped bars at the surface of vertical bars represent the standard deviation, *n* = 3. *YG* yellow gypsum, *NG* natural gypsum, *PGD* Pavagada, *BJP* Baljigapade.

Based on the uptake of micronutrients and silicon by groundnut as well as their available content in the soil, the value of plant available nutrient (PAN) recovery coefficient was calculated and presented in Fig. 4. Analysis of variance showed that different treatments, locations and their interactions had significant effect on PAN recovery coefficient of micronutrients. PAN recovery coefficient of micronutrients was found to be significantly varied among different treatments at Pavagada. Significantly higher PAN recovery coefficient of Fe (0.19), Zn (0.11) and Cu (0.11) was recorded with basal + split application of 625 kg YG ha^−1^, while for Mn (0.38) it was recorded with basal + split application of 500 kg YG ha^−1^. In Baljigapade, basal + split application of 625 kg YG ha^−1^ resulted significantly higher PAN recovery coefficient of Zn (0.05) and Cu (0.05), and Fe (0.08) in 2018 and 2019, respectively. Regardless of the location, higher PAN recovery coefficient values of micronutrients were observed with high rate of YG application, while lower PAN recovery coefficient values were observed with NG application. Very low PAN recovery coefficient values of micronutrients (PAN recovery coefficient < 1.0) indicated that soils of all three locations were sufficient for covering nutritional needs of groundnut crop.

**Figure 5:**
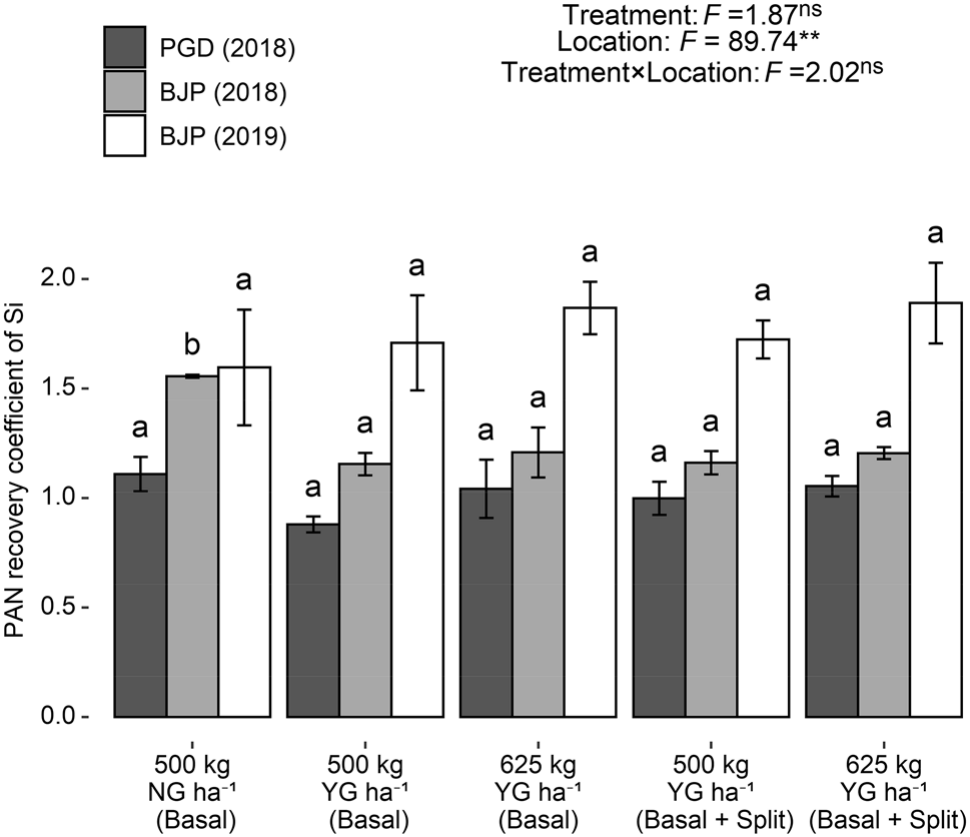
Plant available nutrient (PAN) recovery coefficient of Si in groundnut under different YG treatments. F value and significance level (**P < 0.01, *P < 0.05 and ^ns^*P* ≥ 0.05). Values followed by a different letter within the same location are significantly different at *P* < 0.05 probability level. Capped bars at the surface of vertical bars represent the standard deviation, *n* = 3. *YG* yellow gypsum, *NG* natural gypsum, *PGD* Pavagada, *BJP* Baljigapade.

For silicon, different treatments and its interaction with location had no significant effect on PAN recovery coefficient of silicon, but found to be significant due to location (P < 0.01) (Fig. 5). PAN recovery coefficient of Si was not varied significantly among different YG receiving treatments, except at Baljigapade in 2018. All the YG receiving treatments recorded lower PAN recovery coefficient value than NG receiving treatment. Higher values of PAN recovery coefficient for silicon (PAN recovery coefficient > 1.0) showed that quantity of this beneficial element in soil was too low to cover plant nutritional requirements.

### Micronutrient and silicon uptake by haulm and kernel

Uptake of micronutrients by haulm and kernel of groundnut was found to be significantly affected by different treatments and locations (P < 0.01). Nonetheless, treatment and location interaction had no significant effect on micronutrients uptake by kernel of groundnut (Table 5). Uptake of micronutrients by both haulm and kernel of groundnut largely increased in YG applied treatments, comparing with control treatment. Among different treatments, uptake of Fe, Mn, Zn and Cu by haulm of groundnut was in the range 1562.38–3915.44 g ha^−1^, 237.08–691.86 g ha^−1^, 82.69–252.32 g ha^−1^ and 70.74–193.11 g ha^−1^, respectively. Irrespective of the locations, treatment received 625 kg YG ha^−1^ as basal + split recorded significantly higher micronutrient uptake, while lower micronutrient uptake was recorded with 500 kg NG ha^−1^. Total uptake of Fe, Mn and Cu by groundnut were significantly and positively correlated with availability of Fe (*r* = 0.93*), Mn (*r* = 0.91*) and Cu (*r* = 0.89*), respectively (see Supplementary Fig. S1 online). In general, application of 500 and 625 kg YG ha^−1^ at different time had no significant effect on micronutrient uptake. Among the locations, significantly higher uptake of micronutrients was observed at Baljigapade in 2019 followed by Baljigapade and Pavagada in 2018.

**Table 5:** Effect of different sources, time and rate of application of gypsum on uptake of micronutrients and silicon by haulm and kernel of groundnut at harvest.

Locations and year of experiment	Gypsum sources	Time and rate of application (kg ha^−1^)		Haulm					Kernel				
		Basal	Split	Fe	Mn	Zn	Cu	Si	Fe	Mn	Zn	Cu	Si
				(g ha^−1^)					(g ha^−1^)				
Pavagada 2019	NG	500	–	1782.37 b	237.08 b	82.69 b	70.74 c	35.66 b	250.10 c	39.45 a	27.63 c	18.38 a	4.96 a
	YG	500	–	2057.64 ab	262.44 b	122.94 a	90.06 ab	37.72 b	291.34 bc	38.84 a	28.90 bc	20.86 a	5.31 a
		625	–	2462.22 a	310.63 a	109.60 ab	95.03 a	46.29 a	326.60 ab	42.32 a	33.85 b	22.15 a	7.09 a
		250	250	2062.57 ab	254.88 b	121.71 a	84.95 b	46.71 a	304.74 bc	36.61 a	29.81 bc	18.21 a	5.08 a
		312.5	312.5	2579.63 a	312.14 a	133.61 a	96.55 a	48.87 a	341.55 a	43.72 a	38.82 a	21.80 a	6.37 a
Baljigapade 2018	NG	500	–	1846.81 c	472.77 b	206.01 c	82.62 b	36.15 bc	264.77 c	55.59 a	21.82 b	17.52 a	5.29 a
	YG	500	–	2348.77 bc	484.74 b	207.12 c	98.24 ab	34.22 c	363.88 c	52.25 a	43.15 a	24.20 a	6.25 a
		625	–	2908.89 a	534.87 ab	232.63 ab	101.73 a	39.73 ab	548.27 ab	60.73 a	38.71 a	28.19 a	7.27 a
		250	250	2833.22 ab	515.90 b	211.90 bc	109.19 a	37.48 bc	405.17 bc	51.06 a	40.22 a	24.80 a	5.78 a
		312.5	312.5	3254.98 a	605.73 a	240.39 a	112.55 a	42.75 a	580.86 a	58.40 a	45.44 a	25.54 a	6.89 a
Baljigapade 2019	NG	500	–	1562.38 d	521.52 b	160.82 b	139.08 c	33.81 b	331.52 b	52.26 bc	54.63 b	21.13 c	4.04 d
	YG	500	–	2712.25 c	516.64 b	188.28 b	144.11 c	38.12 ab	351.43 b	51.63 c	54.46 b	20.97 c	4.98 cd
		625	–	3091.30 b	595.31 b	198.72 b	164.60 b	41.19 a	465.09 a	67.53 ab	65.60 ab	27.24 ab	6.30 b
		250	250	3045.97 b	572.35 b	175.33 b	167.93 b	39.53 a	381.08 b	54.07 bc	60.91 b	24.68 bc	5.24 c
		312.5	312.5	3915.44 a	691.86 a	252.32 a	193.11 a	43.22 a	502.96 a	78.91 a	75.92 a	30.46 a	7.39 a
*F* value	Treatments			28.21**	14.26**	13.18**	16.71**	14.11**	15.27**	4.15*	8.45**	4.42**	4.62**
	Locations			16.53**	239.58**	146.90**	237.93**	9.79**	15.67**	21.37**	80.53**	5.00*	11.11**
	Treatments × locations			2.39*	1.09ns	2.36*	2.38*	1.36ns	1.61ns	1.12ns	1.57 ns	0.86ns	1.89ns

F value and significance level (**P < 0.01, *P < 0.05 and ^ns^P ≥ 0.05). Values followed by a different letter within the same column and the same year are significantly different at P < 0.05 probability level.

*YG* yellow gypsum, *NG* natural gypsum.

Different treatments and locations significantly affected the Si uptake by haulm of groundnut (P < 0.01). Nevertheless, treatment and location interaction had no effect on Si uptake by haulm and kernel of groundnut (Table 5). YG application at different rate and time had significant effect on Si uptake by haulm of groundnut and higher Si uptake was observed with basal + split application of 625 kg YG ha^−1^. Further, all YG receiving treatments had significantly higher Si uptake than NG receiving treatment except treatment receiving 500 kg YG ha^−1^ as basal. Application of same level of YG as basal alone and basal + split was found to be at par with each other. The Si uptake by kernel was not significantly varied among treatments at Pavagada and Baljigapade in 2018, however, there was significant variation at Baljigapade in 2019. Higher and lower Si uptake by groundnut kernel was observed with basal + split application of 625 kg YG ha^−1^ (7.39 g ha^−1^) and basal application of 500 kg NG ha^−1^ (4.04 g ha^−1^), respectively.

### Economic analysis

Compared to control where 500 kg NG ha^−1^ was applied, all the YG treatments achieved maximum returns in producing maximum gross income and benefit: cost ratio (Table 6). Maximum gross return (2420.56 US$ ha^−1^) and benefit: cost ratio (3.00) was achieved by basal + split application of 625 kg YG ha^−1^. The lowest gross income (2169.72 US$ ha^−1^) and benefit: cost ratio (2.73) was recorded in treatment where 500 kg NG ha^−1^ was applied as basal.

**Table 6:** Economic analysis of groundnut growing under different YG rates and application timings based on current prices (Pooled data).

Gypsum sources	Time and rate of application (kg ha^−1^)		Total cost (US$ ha^−1^)	Gross income (US$ ha^−1^)	Net income (US$ ha^−1^)	Benefit: cost ratio
	Basal	Split				
NG	500	–	581.37	2169.72	1588.35	2.73
YG	500	–	595.37	2217.40	1622.03	2.72
	625	–	604.12	2335.52	1731.40	2.86
	250	250	595.37	2246.24	1650.86	2.77
	312.5	312.5	604.12	2420.56	1816.44	3.00

Current price (US$): Groundnut kernel—$0.81/kg, Groundnut haulm—$0.013/kg, Yellow gypsum—$0.067/kg, Natural gypsum—$0.040/kg.

*NG* natural gypsum, *YG* yellow gypsum.

## Discussion

During 2019 at Baljigapade, overall rainfall was eight and twofold higher than Pavagada and Baljigapade in 2018, respectively, which might have induced the release of divalent cations both from exchange sites and, dissolution of gypsum sources and other minerals for better nutrition^31^ and thereby resulted in the higher yield at Baljigapade in 2019. Irrespective of the locations, the mean monthly atmospheric temperature was found to be optimum for vegetative (between 25 and 28 °C) and reproductive stage (between 22 and 25 °C) of groundnut^32^. Regardless of the location, the sunshine hours day^−1^ ranged from 10.16 to 11.86 h day^−1^ during vegetative and peg initiation stage, while at the reproductive stage it recorded < 11 h day^−1^. Groundnut being a short-day plant, its flowering and peg initiation are not affected when sunshine hours day^−1^ is below 11. These results are in agreement with previously observed higher number of flowers opening in groundnut with 10 sunshine hours day^−1^ than 16 h day^−1^^33^.

Gypsum source used in the present study supplies Fe, Mn, Zn, and Si along with Ca and S. Additional supply of these nutrients from YG might have resulted in higher plant height and number of branches^19^. Fe and Zn play a pivotal role in regulating multiple biochemical reactions in plants thereby resulting in a significant increase in growth parameters with their application as YG to the soil^34^. Although Si is not an essential element for plant growth and development, but positive and significant effect of Si on plant growth parameters of various cereal^35^ and oil seed crops^18,36^ has been widely reported. Improved Si concentration in soil solution as a result of Si fertilization was efficient to improve other mineral nutrition absorption, thus leading to a beneficial effect on growth and development of groundnut^37^. Since YG is produced from calcium silicate slag its application also supplies Ca, S and P which also had a positive effect on the growth of maize, rice and groundnut^19,38,39^. In all three locations, basal + split application of 625 kg YG ha^−1^ recorded significantly higher plant height and the number of branches. Similarly, Kannan et al.^10^ recorded higher plant height and number of branches of groundnut with split application of 400 kg gypsum ha^−1^ (200 kg as basal and 200 kg as top dressing).

Application of YG resulted in more consistent and significant changes in yield and other associated yield components of groundnut compared to NG application (Table 3). Increase in pod and haulm yield of groundnut with YG application was mainly attributed to increased yield components and also due to higher availability of Ca, S, P, micronutrients and Si in the soil^38,40^. There is positive and significant correlation between pod and haulm yield of groundnut and total micronutrients and Si uptake. This indicates that increasing concentration of micronutrients and Si in soil as result of YG application could promote their availability and uptake consequently pod and haulm yield of groundnut. The significant difference in the pod and haulm yield among gypsum sources mainly due to their varied elemental composition, as YG additionally supplies Fe, Zn, P and Si along with Ca and S. Fe and Zn being cofactors of various enzymes and proteins, plays significant role in regulating several physiological and metabolic processes of plants thereby resulting in better yield with their application as YG^41^. Increase in yield of groundnut with Si supply as YG mainly attributed to pH adjustment in the soil^42^ consequently resulted in higher acquisition of macro and micronutrients from soil to plant. A study by Liang et al.^43^ revealed that supply of Si through slags or Si mineral ores indirectly promotes the crop growth by increasing lodging resistance and biotic and abiotic stress resistance. Studies on application of Si to pulse crops mainly evidenced increase in yield by increase in the number of pods plant^−1^, number of seeds in the pod and seed weight^36^. Higher pod and haulm yield and yield components of groundnut due to basal + split application of YG can be mainly attributed to continuous availability of Ca, S, Si and micronutrients for complete lifecycle of the crop^10^. Therefore, basal + split application of YG at 625 kg ha^−1^ could be considered as best source, rate and time of application to obtain higher groundnut yield.

Results on pH of post-harvest soil demonstrated that YG application had a higher impact on soil pH than NG. This finding is not surprising, since YG is a synthetic material produced from steel industry slag which have high lime potential (87.48% CaCO_3_)^44^. A similar observation was noticed by previous studies with the application of slag material to wheat and rice crop at the rate of 10 Mg ha^−1^ and recorded significantly higher pH in the post-harvest soil^45,46^. Dissolution of slag materials are favored by acidic soil conditions^47^ consequently it releases the soluble Si and OH^−^ ions which causes an increase in post-harvest soil pH. The differential effect of gypsum sources on pH mainly attributed to their lime potential and source of production. For instance, application of YG produced from alkaline LD-slag resulting greater change in post-harvest soil pH than naturally mined gypsum. Application of gypsum sources increased the EC of post-harvest soils and which could be mainly attributed to relatively higher proportion of Ca and SO_4_^2−^ in soils after harvest of groundnut^48^.

Increasing levels of YG significantly increased the micronutrient availability in post-harvest soils of all three locations. This effect was expected because the gypsum source (YG) used in these experiments contains micronutrient elements^44^. The significant difference among gypsum sources was mainly due to their varied elemental composition. Greater positive effect of YG on DTPA-extractable micronutrients content in soil was very well noticed at Baljigapade experimental sites than Pavagada experimental site and these contrasting results could be due to variation in increasing pH at all three locations. Positive effect of YG on micronutrient availability is contradicting to what was expected, as their availability decreases with increasing soil pH^49^. This finding could simply be due to pH, since it did not increase beyond 7.5 in all three locations, except with higher levels of YG at Pavagada in 2018. Although YG contained small amount of Cu and Mn but its application increased the availability of these nutrients in post-harvest soils which could be due to the increase in concentration of Ca and Mg in soil as consequent of YG application^50^. Si fertilization through slag type fertilizer significantly increased the available Fe in the soil after rice harvesting^46^. Application of slag proved to significantly increase the Fe availability^51^, and therefore slag and fertilizer material produced from slag containing considerable amounts of micronutrient could be a promising and inexpensive source of micronutrients to alleviate their deficiency. Application of 50% of YG rate at peg initiation stage enables groundnut crop to take up the micronutrients more quickly and efficiently, thereby significantly increase the DTPA-extractable micronutrients with basal + split application of YG over basal application^55^.

Yellow gypsum application significantly increased both CC-Si and AA-Si in all three locations. Two factors may account for this observation. First, application of YG significantly increased the soil pH after groundnut harvesting, which consequently increased the plant-available Si in the post-harvest soil. There is positive relation between pH and plant available Si and increasing pH (up to pH 9.8) increase the proportion of total Si in soil solution^52^. Second, gypsum source used in this study contains Si element as one of its constituents. Application of the same rate of YG and NG had a different effect on plant-available Si, which was mainly attributed to their different composition and different effect on post-harvest soil pH^19^. Meanwhile, higher plant-available Si content in Pavagada than Baljigapade location was also attributed to variation in increasing pH with same rate of YG application. Similarly report of direct effect of increased pH on Si availability to rice crop with steel slag-based silicate fertilizer application has been reported by Ning et al.^53^.

Higher PAN recovery coefficient values in YG receiving treatments could be attributed to relatively higher uptake of micronutrients by groundnut due to their higher availability in the soil as compared to treatment receiving NG. Increased content of micronutrients in groundnut haulm and kernel in response to YG application could be due to the release of these nutrients from both the soil and the YG^28^. These findings suggest that micronutrients applications should be given special consideration to maintain soil fertility at an adequate level for sustainable groundnut production. YG supplies Si in a higher proportion which could be attributed to lower PAN recovery coefficient values with its application than NG application.

Significantly higher uptake of micronutrients by haulm and kernel of groundnut mainly ascribed to higher availability of DTPA-extractable micronutrients in post-harvest soil as a result of YG application^40^. Positive and significant correlation between uptake of Fe, Mn and Cu by groundnut and their availability in the soil could be attributed to increased availability of these nutrients in soils with applied YG thereby increasing their uptake by groundnut. The significant difference among gypsum sources mainly attributed to their varied chemical composition, where YG contains a higher percentage of micronutrients than NG, especially Fe (5.41%) and Zn (0.37%)^44^. Studies with slag application as Fe, Mn and Cu to maize crop reported higher uptake these nutrients^51,54^. Higher uptake of micronutrients with basal + split application of YG could be due to continuous availability of these nutrients to groundnut plants throughout lifecycle^55^.

Higher Si uptake in YG received treatments than NG was mainly due to higher Si content in YG (3.41%). Among three locations, Pavagada in 2018 recorded higher Si uptake by haulm and kernel than Baljigapade locations, which mainly ascribed to higher post-harvest soil pH in former location. A strong positive relationship between soluble Si and soil pH has been widely reported^56^. A linear increase in Si uptake with increasing YG rate indicating that the Si in the applied YG was available to the plant and absorbed into the plant tissues. Yang et al.^57^ observed a linear increase in straw Si content with the application of slag material at different rates in rice.

The field scale adaptability of any novel and emerging technique depends upon its economic feasibility. The economic analysis revealed that basal + split application of 625 kg YG ha^−1^ generated maximum net returns and benefit: cost ratio which mainly attributed to higher pod and haulm yield under this treatment. It is very apparent that application of same level of YG as basal alone and basal + split made remarkable difference in net return and benefit: cost ratio (Table 6). Thus, basal + split application of 625 kg YG ha^−1^ could effectively be used to improve economic returns of groundnut.

## Conclusion

The results of present study revealed that application of yellow gypsum significantly improved the soil pH, availability of micronutrients, silicon and their uptake by groundnut when compared to natural gypsum. Among different time and rate of yellow gypsum application, basal + split application of 625 kg ha^−1^ of YG increased available nutrients in the soils as well as their uptake, growth and yield of groundnut. Further, application of yellow gypsum as a split dose resulted in higher economic returns than basal application of yellow gypsum and natural gypsum. Therefore, application of yellow gypsum as basal + split at the rate of 625 kg ha^−1^ may be considered as a best strategy to enhance overall productivity of groundnut by improving soil and crop nutritional status. In comparison to natural gypsum, yellow gypsum additionally supplies higher iron, zinc, and silicon along with calcium and sulphur. Hence, it could be a cost effective and potential source for alleviation of these nutrient deficiencies in soils and also could be a better alternative for natural gypsum.

## Supplementary Information

Supplementary Figure S1.
