# Supplementary material for: Influence of yellow gypsum on nutrient uptake and yield of groundnut in different acid soils of Southern India
Source: Sci Rep. 2022 Apr 4;12:5604. doi: 10.1038/s41598-022-09591-1 (PMC8979954; doi:10.1038/s41598-022-09591-1)
Supplement: Supplementary file 1 — Supplementary Figure S1. [file 41598_2022_9591_MOESM1_ESM.pdf]

Influence of yellow gypsum on nutrient uptake and yield of groundnut in different acid soils of Southern India

Laxmanarayanan M[1], Prabhudev Dhumgond[1], Shruthi[1], Jahir Basha C R[2], Prakash Nagabovanalli B[1*]

[1] Plant Nutrition Laboratory, Department of Soil Science and Agriculture Chemistry, University of Agricultural Sciences, GKVK, Bengaluru - 560065, Karnataka, India.

[2] Agricultural Research Station, Pavagada, Tumkur - 561202, Karnataka, India.

*Corresponding author: nagabovanalliprakash@rediffmail.com

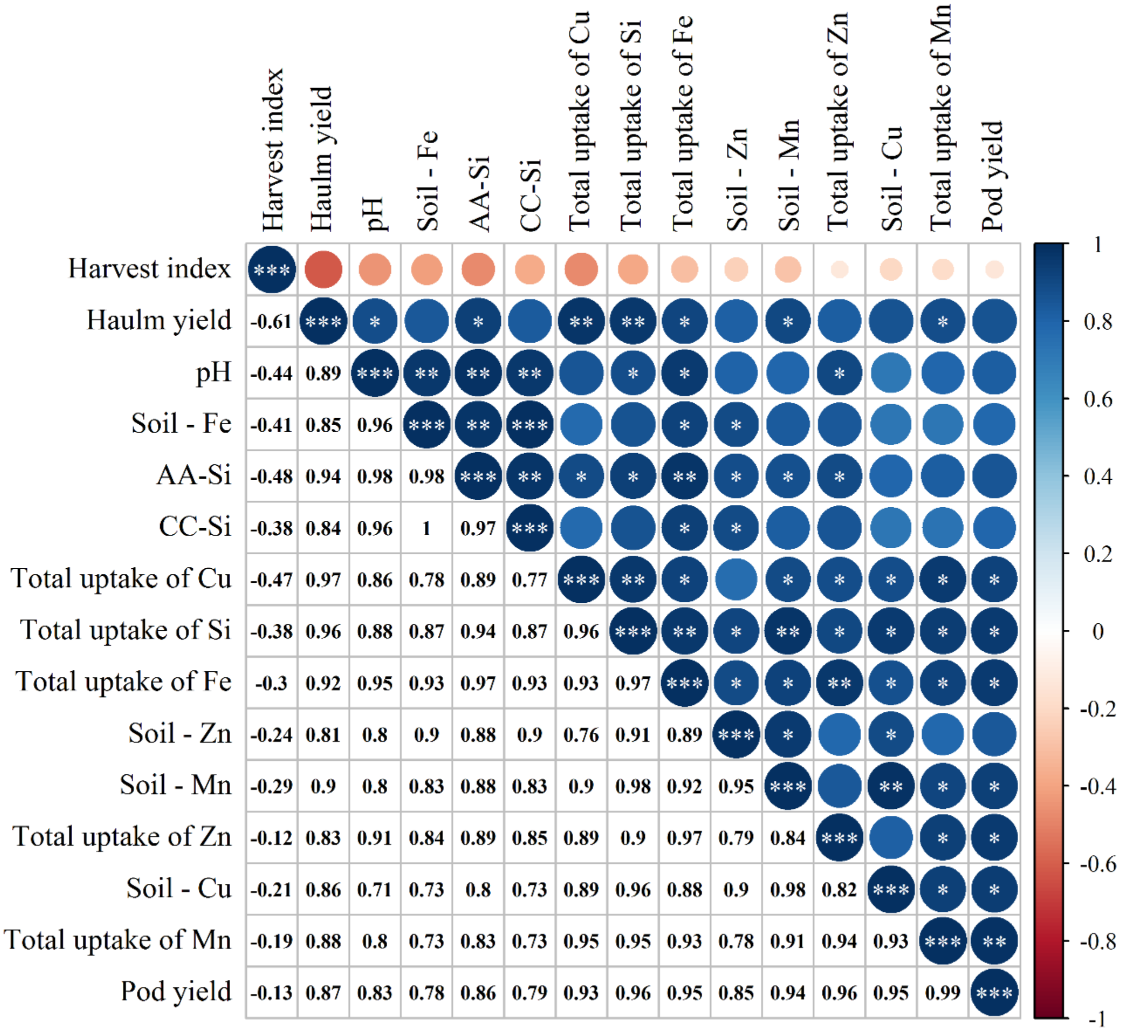

Fig. S1. Pearson's correlation coefficient between available micronutrient and Si at harvest and their total uptake by groundnut, pod and haulm yield, harvest index and pH.CC-Si - $CaCl_2$ extractable Si, AA-Si - Acetic acid extractable Si.
